# Effects of Saffron Extract (Affron^®^) with 100 mg/kg and 200 mg/kg on Hypothalamic–Pituitary–Adrenal Axis and Stress Resilience in Chronic Mild Stress-Induced Depression in Wistar Rats

**DOI:** 10.3390/nu15234855

**Published:** 2023-11-21

**Authors:** Chae-Young Kim, Kayoung Ko, Seo-Hee Choi, Miri Jo, Jinhye Kim, Sunmi Yoon, Isaac Jinwon Yi, María Inés Morán-Valero, Min-Young Kwon, Johann Sohn, Sun-Shin Yi

**Affiliations:** 1 BK21 Four Program, Department of Medical Science, Soonchunhyang University, Asan 31538, Republic of Korea; kcybada8844@sch.ac.kr; 2 Department of Biomedical Laboratory Science, College of Medical Sciences, Soonchunhyang University, Asan 31538, Republic of Korea; gurqls0404@naver.com (K.K.); suhhee1021@naver.com (S.-H.C.); jml2213@naver.com (M.J.); 3 Central Lab., iCONNECTOME Co., Ltd., Cheonan 31168, Republic of Korea; adjh08@iconnectome.com (J.K.); sycshm0227@iconnectome.com (S.Y.); 4 Department of Cognitive Science, University of California, San Diego, CA 92093, USA; jiyi@ucsd.edu; 5 Pharmactive Biotech S.L.U., 28049 Madrid, Spain; ines.moran@pharmactive.eu; 6 Hyundai Bioland Co., Ltd., Ansan 15407, Republic of Korea; mykwon0217@hyundaibioland.co.kr (M.-Y.K.); jsohn@hyundaibioland.co.kr (J.S.)

**Keywords:** saffron extract, Affron^®^, hypothalamic–pituitary–adrenal axis, unpredictable chronic mild stress, depression, stress resilience, stress hormones, neuroplasticity

## Abstract

Stress-related symptoms are a global concern, impacting millions of individuals, yet effective and safe treatments remain scarce. Although multiple studies have highlighted the stress- alleviating properties of saffron extract, the underlying mechanisms remain unclear. This study employs the unpredictable chronic mild stress (CMS) animal model to investigate the impact of a standardized saffron extract, Affron^®^ (AFN), on hypothalamic–pituitary–adrenal (HPA) axis regulation and neuroplasticity in Wistar rats following repeated oral administration. The research evaluates AFN’s effects on various stress-related parameters, including hypothalamic gene expression, stress hormone levels, and the sucrose preference test. In animals subjected to continuous unpredictable CMS, repetitive administration of AFN at doses of 100 mg/kg and 200 mg/kg effectively normalized HPA axis dysregulation and enhanced neuroplasticity. Increased concentrations of AFN demonstrated greater efficacy. Following AFN oral administration, adrenocorticotropic and corticosterone hormone levels exhibited significant or nearly significant reductions in comparison to subjects exposed to stress only. These changes align with the alleviation of stress and the normalization of the HPA axis. These findings elucidate AFN’s role in stress mitigation, affirm its health benefits, validate its potential as a treatment for stress-related symptoms, confirm its physiological effectiveness, and emphasize its therapeutic promise.

## 1. Introduction

Contemporary global challenges, ranging from socio-economic perturbations to unexpected pandemics like COVID-19, emphasize the growing imperative to understand and address the multifaceted impact of chronic stress on mental health [1,2]. Prolonged exposure to diverse stressors, especially those that are unpredictable, triggers a cascade of physiological and psychological responses [3]. These responses often lead to the onset of depressive symptoms, anxiety, and various cognitive and emotional impairments [4,5]. 

Stress resilience is the organism’s ability to reestablish its fundamental psychological and physiological equilibrium in the face of stress, skillfully managing stressors and adapting constructively [6]. This trait is crucial as it reduces the risk of chronic health issues by enabling a rapid return to a state of balance post-stress [6,7]. Allostasis is intimately connected to this concept, referring to the active maintenance of stability via physiological mechanisms [6]. It is a process involving the autonomic nervous system, the hypothalamic–pituitary–adrenal (HPA) axis, and systems governing cardiovascular, metabolic, and immune functions [8]. These systems collectively respond to internal and external stressors by producing mediators such as cortisol, aiding the body’s adaptation to various challenges. However, when faced with unceasing stressors, the body may be forced to adjust to a new, often more taxing set point—a phenomenon termed as establishing a ‘new normal’ [9]. This recalibration, while adaptive, incurs what is known as allostatic load [9]. This concept describes the cumulative cost of adaptation, manifesting as physiological wear and tear resulting from continuous or intense engagement of adaptive systems [6]. Such a state, derived from sustained physiological responses to psychosocial or physical stressors, has been identified as having potential adverse health implications, highlighting the delicate balance between necessary adaptation and the potential for harmful overactivation of these systems. Related to this, central to these stress-induced alterations is the hypothalamic–pituitary–adrenal (HPA) axis [6,10], a vital neuroendocrine system intricately involved in orchestrating the body’s adaptive responses to stress [6,11]. Natural remedies have historically played a pivotal role in alleviating the symptoms of mental distress [12]. Saffron (*Crocus sativus* Linn), a distinguished spice with a rich history of medicinal use, exemplifies this approach [13]. Its therapeutic benefits have been discussed for centuries as essential components of traditional healing practices. Scientific investigations have unveiled the molecular mechanisms underlying its therapeutic potential [14]. Among its bioactive constituents, crocin, safranal, crocetin, and picrocrocin mediate a plethora of physiological effects [15]. Extensive research highlights saffron’s medicinal properties, showcasing its anti-inflammatory [16,17], anti-tumor [18], anti-diabetic [19], and anti-obesity [20] activities. Numerous studies have confirmed the efficacy of saffron extract and its constituents in alleviating stress [13,21,22]. Of particular interest is the standardized saffron extract Affron^®^ (AFN). Preliminary clinical and experimental investigations have consistently indicated AFN’s significant effectiveness in modulating mood during eight weeks in adults with persistent depression who were given a saffron extract (AFN, 14 mg b.i.d.), alleviating stress and enhancing mental well-being across diverse populations [23]. Analyses have shown that a daily dose of 28 mg considerably reduces mood disturbances, stress, and anxiety-related symptoms [20]. AFN has the potential to mitigate the severity of depressive symptoms, akin to the effects of tricyclics, selective serotonin reuptake inhibitors, and selective noradrenaline reuptake inhibitors [24,25]. Furthermore, an eight-week AFN regimen for adolescents (12–16 years) with mild-to-moderate anxiety and/or depressive symptoms demonstrated substantial improvement in their internalized symptoms [26]. 

Despite the wealth of clinical evidence supporting AFN’s efficacy in managing depression and anxiety symptoms, elucidating its precise mechanism of action warrants further study. Our previous animal behavioral assays assessing anxiety and depression, including the elevated plus maze, forced swim, and sucrose preference tests, have revealed that AFN-treated animals (200 mg/kg) exhibit behaviors indicative of anhedonia and depression mitigation [27]. For example, increased consumption of sugar solutions and improved specific escape responses have been observed in forced swim tests. Building on the previously affirmed beneficial outcomes of AFN, we adjusted the dosage to 100 and 200 mg/kg to further investigate the mechanism by which AFN delivers its anti-depressant impact within the organism. Therefore, this present study sets out to comprehensively elucidate the nuanced effects of repeated oral administration of AFN on the modulation of the HPA axis, particularly in the context of unpredictable chronic mild stress (CMS)-induced depression in animals. To assess the resilience of the hypothalamic–pituitary–adrenal (HPA) axis’s normal negative feedback mechanism in response to stress, we measured arginine vasopressin (AVP) expression in the hypothalamic paraventricular nucleus (PVN) and supraoptic nucleus (SON), glucocorticoid receptor (GR) expression in the PVN, and corticotropin-releasing factor (CRF) in the hypothalamus. Additionally, we used a brain-derived neurotrophic factor (BDNF) antibody to assess neuroplasticity alterations in the hypothalamus following AFN administration. BDNF, ubiquitously present in the mammalian brain, plays pivotal roles in neuronal survival, nerve regeneration, synaptic function, and neurogenesis [28]. We also compared differences in blood adrenocorticotropic hormone (ACTH), corticosterone hormone (CORT), and serotonin (5-HT) levels. Conducting research to measure these biomarkers is crucial for understanding the system’s dynamics [29,30,31]. An assessment was conducted to determine whether repeated AFN administration could restore normal sucrose consumption patterns. Comprehensive brain scans were performed to identify the brain regions influenced by AFN. 

In these findings, we aspire to provide a comprehensive insight into the mechanistic interplay between a standardized saffron extract, Affron^®^, and the HPA axis. Our overarching goal is to spotlight the potential of natural remedies, particularly saffron, in bolstering resilience against the detrimental impacts of chronic stress, as evidenced in our Wistar rat model.

## 2. Materials and Methods

### 2.1. Test Substance

The test substance, AFN (sourced from Pharmactive Biotech Products S.L., Madrid, Spain), was accurately weighed and then transferred into a 50 mL centrifuge tube. It was subsequently suspended in distilled water, serving as the designated vehicle, through vortexing. The dosing formulation was then adjusted to achieve the intended concentration (100 or 200 mg/kg/day) using the same vehicle. This formulation was freshly prepared immediately before daily administration using stainless steel zonde (Φ1.2 × 100 mm (18 gauze)), and both the vehicle and test substances were orally administered simultaneously between 10:00 AM and 12:00 PM each day for three weeks, from the second to the fourth week.

### 2.2. Animals and Experimental Groups

For this study, a cohort of 40 male Wistar rats (Orient Bio, Gyeonggi-do, Korea), with individual weights ranging from 200 to 220 g, was employed. The animals were housed under standard conditions of room temperature (22 ± 1 °C) and 60% humidity, with a 12 h light–dark cycle (light:dark cycle from 07:00 AM to 07:00 PM). They were provided a normal chow diet (Envigo, Indianapolis, IN, USA), and their dietary intake was continuously monitored using the BioDAQ^®^ food intake monitoring system (Research Diet, New Brunswick, NJ, USA). The rats were then divided into four distinct experimental groups, each comprising ten animals. The dose volume for each animal was calculated based on their most recently recorded body weight. The experimental group design was as follows:

1. Group 1 (control): Rats in this group received no specific treatment and were solely administered the vehicle.
2. Group 2 (stress-induced): Rats in this group were subjected to unpredictable CMS and subsequently administered the vehicle.
3. Group 3 (AFN 100 mg/kg): Rats in this group were exposed to unpredictable CMS and administered AFN at a dosage of 100 mg/kg (CMS + AFN 100 mg/kg/day).
4. Group 4 (AFN 200 mg/kg): This group consisted of rats that underwent unpredictable CMS and were given AFN at a dose of 200 mg/kg (CMS + AFN 200 mg/kg/day).

To conduct a preliminary assessment, the first two animals from each group were sacrificed to identify the target brain region for the AFN oral administration. The remaining eight animals from each group were utilized for subsequent experiments.

### 2.3. Experimental Models

**Figure 1:**
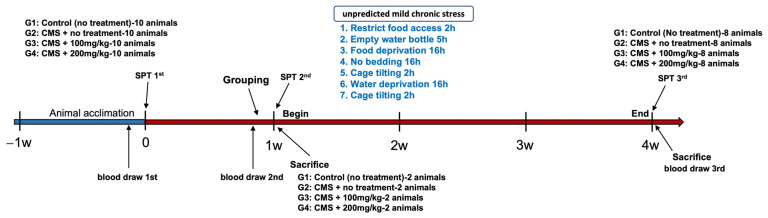
Schematic illustration of the experimental schedule for unpredictable chronic mild stress (CMS) and administration. The experimental schedule spanned four weeks, not inclusive of the animal acclimation process. To assess hormonal alterations, peripheral blood was drawn from the animals (via jugular vein) on three separate occasions. Throughout the experiment, three sucrose preference tests (SPTs) were performed to monitor variations in sucrose preference in relation to the administration of the test substance. CMS was performed using a total of 7 methods.

The experimental protocol spanned a total duration of four weeks, as depicted in Figure 1. During the initial week, only the CMS procedure was conducted. Commencing from the second week and extending through the fourth week, the administration of the designated substances was carried out.

#### 2.3.1. Unpredictable Chronic Mild Stress (CMS)

The CMS model was first proposed by Willner in 1984 [32]. In this study, we made partial modifications to the traditional experimental method (Table 1). Except during periods of deliberate food and water deprivation, all participants had ad libitum access to both food and water, leading to the sucrose preference test.

#### 2.3.2. Sucrose Preference Test (SPT)

Before conducting the sucrose preference test, the animals were not subjected to food or water deprivation. During the adaptation phase, the animals were housed in their native cages and provided with two bottles identical to those used in the test to acclimate them to the test conditions. Subsequently, the rats were simultaneously presented with two identical drinking bottles, both containing a 1% sucrose solution, for a duration of three hours. To eliminate potential place preference effects, the positions of the drinking bottles were alternated after each measurement. The consumption of both water and sucrose solutions was determined by weighing the bottles before and after the tests [27]. The preference for sucrose was calculated as follows:

(1) $$\mathrm{Quantity}\;\mathrm{of}\;\mathrm{sucrose}\;\mathrm{solution}\;=\frac{\mathrm{Volume}\;\mathrm{of}\;\mathrm{sucrose}\;\mathrm{solution}\;\mathrm{consumed}\;}{\mathrm{Total}\;\mathrm{fluid}\;\mathrm{intake}\;}$$

### 2.4. Tissue Processing and Histology

The animals underwent transcardial perfusion with 0.01 M phosphate-buffered saline (PBS; pH 7.4), followed by 4% paraformaldehyde (PFA) in 0.1 M phosphate buffer (PB; pH 7.4). Subsequently, the brains were extracted and post-fixed in the same fixative (4% PFA) overnight at 4 °C. The brain tissues were cryoprotected by immersion in a 30% sucrose solution overnight. Following this, the frozen tissues were serially sectioned at 30 or 40 µm intervals in the coronal plane using a cryo-microtome (Thermo Fisher Scientific, Walldrof, Germany) at −20 °C. These tissue sections were placed in a cryopreservation solution and stored in a refrigerator for future use.

#### 2.4.1. Nissl Staining

For Nissl staining, brain tissues were stained with 1% cresyl violet (Daejung Chemicals & Metals, Siheung, Korea). Slices were incubated in the staining solution for 10 min with consistent agitation. After staining, slices were dehydrated in ethanol ranging from 70% to 100% for one minute each. The samples were air-dried at room temperature for 20 min and then sealed using Eukitt^Ⓡ^ Quick hardening mounting medium (Sigma Aldrich, St. Louis, MO, USA).

#### 2.4.2. Immunohistochemistry (IHC) for AVP, c-fos, and GR

Uniform immunohistochemical procedures were consistently applied across all groups. For the hypothalamic areas (PVN and SON), sections were obtained using Bregma coordinates ranging from −1.08 to −2.04 mm, with a tissue thickness of 30 µm. To identify the brain hypothalamic regions responsive to AVP and GR, tissues were sectioned at 40 µm intervals, starting from the olfactory bulb and ending just before the cerebellum.

To inhibit endogenous peroxidase activity, the sections were incubated in 0.3% hydrogen peroxide (H_2_O_2_) in PBS for 15 min at room temperature. Following incubation, the tissues were rinsed thrice with 0.01 M PBS. Subsequently, the sections were blocked for one hour at room temperature using Universal Block Serum (Sigma-Aldrich, St. Louis, MO, USA). 

Brain sections were incubated overnight at 4 °C with primary antibodies: rabbit anti-AVP (1:300, Phoenix Pharmaceuticals, Bulingame, CA, USA), mouse anti-c-fos (1:300, Santa Cruz Biotechnology, Dallas, TX, USA), and rabbit anti-GR (1:300, Santa Cruz Biotechnology, Dallas, TX, USA). Subsequently, the sections were incubated with biotinylated goat anti-rabbit IgG (H+B) secondary antibodies (Vector Laboratories, Burlingame, CA, USA) for 2 h at room temperature. Following a 30 min incubation with the VECTASTAIN^®^ ABC-HRP kit (Vector Laboratories, Burlingame, CA, USA), visualization was facilitated by staining with 3,3’-diaminobenzidine (DAB) supplemented with 0.03% H_2_O_2_ in a 0.1M Tris-HCl buffer (pH 7.2). The stained sections were mounted onto silane-coated slides and allowed to dry overnight at room temperature. Once completely dry, the sections were sealed using Eukitt^®^ Quick hardening mounting medium (Sigma-Aldrich, St. Louis, MO, USA). 

### 2.5. Quantification of BDNF and CRF Expression in Hypothalamic Tissues using Western Blot

The protein expression levels of brain-derived neurotrophic factor (BDNF) and corticotropin-releasing factor (CRF) in the hypothalamus were determined by Western blotting, using primary antibodies specific to BDNF and CRF.

Hypothalamic tissues were carefully dissected from the skulls of Wistar rats and immediately homogenized in a lysis buffer (iNtRon Biotechnology, Seongnam, Korea). The protein concentrations were quantified using the BCA kit (iNtRon Biotechnology, Seongnam, Korea). Protein samples were then subjected to electrophoresis using a Novex 4%–12% Tris-Glycine Mini Protein Gel (XP04205, Thermo Fisher Scientific, Carlsbad, CA, USA) and electro- transferred onto PVDF membranes (PR04574, Merck Millipore, Carrigtwohil, Cork, Ireland). The membranes were blocked with 5% (*w/v*) bovine serum albumin (BSA) (GeneAll Biotechnology, Seoul, Korea) in TBST (Biosesang, Yongin, Korea) for one hour at room temperature. Subsequently, membranes were probed with primary antibodies, rabbit anti-BDNF (1:3000, Proteintech, Rosemount, IL, USA), rabbit anti-CRF (1:200, ABBIOTEC, San Diego, CA, USA), and rabbit anti-GAPDH (1:10,000, Cell Signaling, Danvers, MA, USA), and incubated overnight at 4 ℃. After washing, the membranes were incubated with horseradish peroxidase-conjugated secondary antibodies (1:2000; Vector Laboratories, Bulingame, CA, USA) for 2 h at room temperature. Protein bands were visualized using ECL Western blotting detection reagents (RPN2232; GE Healthcare, Buckinghamshire, UK), and images were captured using a Chemiluminescence Bioimaging Instrument (CELLGENTEK, Daejeon, Korea).

### 2.6. Measurement of Plasma ACTH, CORT, and 5-HT Levels using ELISA Assay

The blood samples were collected via the jugular vein of the animals between 07:00 and 08:00. To quantify plasma levels of ACTH, CORT, and 5-HT, we employed enzyme-linked immunosorbent assay (ELISA) kits provided by MyBioSource, Inc. (San Diego, CA, USA). The assays were carried out in strict accordance with the manufacturer’s instructions. Briefly, blood samples were centrifuged to obtain plasma, which was then aliquoted and stored at −80 °C until further analysis. For ACTH and 5-HT measurements, each sample was introduced into respective pre-coated ELISA plates, followed by the addition of a biotinylated detection antibody. After an incubation period, plates were thoroughly washed to remove unbound substances. Subsequently, avidin linked to horseradish peroxidase (HRP) was introduced. In the case of CORT, samples were added to pre-coated plates with the target antigen. During this reaction, the target in the standard sample competed with a consistent amount of the target on the solid-phase support for binding sites on the biotinylated detection antibody specific to that target. After eliminating excess conjugate and unbound samples or standards through washing, HRP-streptavidin (SABC) was dispensed into each microplate well and incubated. Following another incubation period, a substrate solution was introduced, resulting in a color change proportionate to the hormone quantity in the sample. The reaction was then terminated, and the optical density of each well was measured at 450 nm using a microplate reader. Hormone concentrations in the blood samples were calculated using a standard curve derived from reference standards provided in the kit. 

All samples were assayed in duplicate, and the average reading was utilized for data analysis. The intra- and inter-assay coefficients of variation, as well as the sensitivity of the kits, were determined in accordance with the specifications provided by MyBioSource.

### 2.7. Ethical Consideration

All experimental protocols underwent review and approval by the Institutional Animal Care and Use Committee (IACUC) of Soonchunhyang University (approval number: SCH22-0141). All animal experiments described in this study were conducted in full compliance with the committee’s guidelines. 

### 2.8. Image Analysis

Ultra-high-resolution images were acquired using a MoticEasyScan One Slide Scanner (Motic, Xiamen, China). Positive cell counts were determined using Image J (v1.53, National Institutes of Health, Bethesda, MD, USA), or the area of the corresponding region was estimated and used as data. Statistical analyses were performed using Prism 9 (GraphPad, San Diego, CA, USA), and statistical comparisons were conducted using unpaired Student’s *t*-test and one- or two-way ANOVA test followed by Dunnett’s post hoc test. Only results with *p* < 0.05 are considered significant, and results are presented expressed as mean ± SEM. 

## 3. Results

### 3.1. Changes in Body Weight, Food, and Water Intake

**Figure 2:**
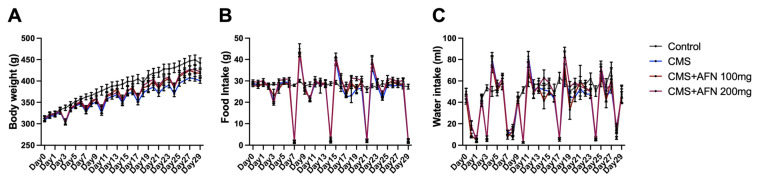
Changes in body weight, food, and water intake. (**A**) The graph illustrates weight fluctuations throughout the study duration. Apart from the control group, the other three groups demonstrated weight loss corresponding with repeated CMS schedules. Despite this trend, weight gain was noticeable in both groups treated with Affron^®^ (AFN) in conjunction with CMS; however, the change was not statistically significant. Over the course of the study, the two AFN-administered groups displayed a more pronounced trend toward weight recovery compared to the CMS-only group. (**B**) The second graph represents variations in food consumption during the study period. Excluding shifts in food intake resulting from imposed food or water restrictions, no significant changes in daily food intake were detected. Following periods of food restriction, a transient surge in food consumption, higher than typical levels, was observed. (**C**) Daily water consumption of the animals was consistently monitored. After water deprivation aligned with the CMS protocol, a brief spike in water consumption was noted. Outside of this particular occurrence, no distinctive alterations in daily water intake among the animals were identified. The error bar represented the mean ± standard error mean (SEM).

Animals subjected to CMS displayed a propensity for weight loss, but the administration of AFN appeared to support a gradual weight regain. However, the groups observed no significant differences in food and water consumption, as illustrated in Figure 2.

### 3.2. Sucrose Preference Test (SPT)

**Figure 3:**
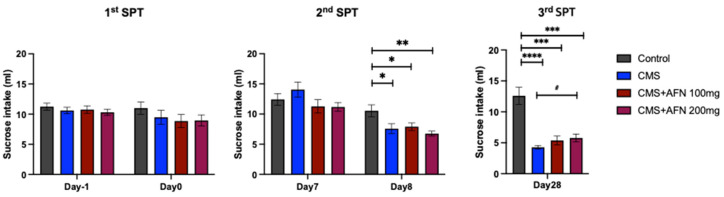
Before entering the first CMS schedule, animals acclimatized to the sucrose preference test (SPT) program exhibited no substantial differences between the groups (1st SPT). However, sucrose intake, as measured one-week post CMS administration, was significantly diminished in all groups subjected to CMS compared to the control group (2nd SPT). Subsequently, with three additional weeks of CMS, the sucrose intake of the groups subjected to CMS continued to decrease. Nevertheless, a notable increase in sucrose intake was observed in the CMS + AFN 200 mg/kg group compared to the CMS group. ****, *p* < 0.001; ***, *p* < 0.005; **, *p* < 0.01; *, *p* < 0.05 vs. control, ^#^, *p* < 0.05 vs. CMS. The error bars represented the mean ± SEM.

The animals displayed a notable reduction in sucrose intake one week post initiation of CMS. However, AFN administration did not lead to a recovery in sucrose intake analogs by the end of the experiment compared to the control group. Intriguingly, the CMS + AFN 200 mg/kg group exhibited a significant increase in sucrose consumption compared with the CMS group, as illustrated in Figure 3. Repeated-measures one-way ANOVA found an overall interaction: Day 1 (F (3,36) = 0.47, *p* = 0.7074), Day 0 (F (3,36) = 0.89, *p* = 0.4544), Day 7 (F (3,36) = 1.68, *p* = 0.1886), Day 8 (F (3,36) = 4.86, *p* < 0.01), Day 28 (F (3,28) = 19.87, *p* <0.001).

### 3.3. Immunohistochemistry for AVP, and GR in the Hypothalamic Area

#### 3.3.1. Expression of AVP in the Hypothalamic PVN and SON Regions

**Figure 4:**
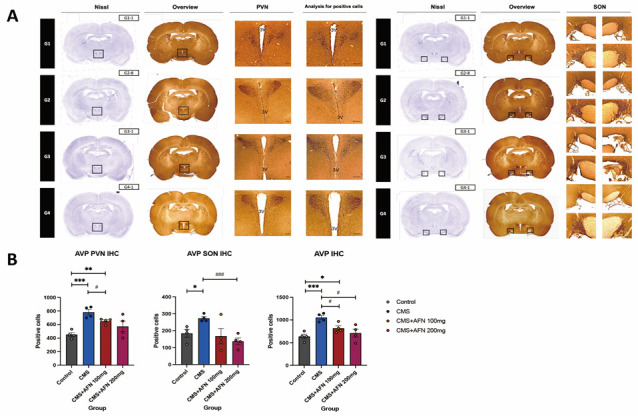
Quantification of arginine vasopressin (AVP)-positive cells in the hypothalamic PVN and SON regions. In the hypothalamic paraventiricular nucleus (PVN) and supraoptic nucleus (SON) regions, the number of AVP-positive cells was assessed. The specific coordinates for observing AVP-positive cells were determined through Nissl staining. (**A**) Relative to the control group, the CMS group exhibited a marked increase in the number of AVP-positive cells in both PVN and SON regions. (**B**) As the dose of AFN increased, there was a notable decline in AVP expression. Particularly in the CMS + AFN 200 mg/kg group, the number of AVP-expressing cells in both the PVN and SON was significantly lower than in the CMS group. Moreover, in both PVN and SON regions, all groups treated with AFN showed a significant reduction in AVP-positive cells compared to the CMS group. ***, *p* < 0.005; **, *p* < 0.01; *, *p* < 0.05 vs. control, ^###^, *p* < 0.005; ^#^, *p* < 0.05 vs. CMS. The error bars represent mean ± SEM. Scale bar = 100 μm.

Using IHC, the number of cells displaying a positive reaction to the AVP antibody in both the PVN and SON were enumerated and compared across the groups. The CMS group exhibited a significantly higher count than the other three groups. In both the PVN and SON regions, the CMS + AFN 100 mg/kg and CMS + AFN 200 mg/kg groups showed a significant reduction compared to the CMS group. However, in the PVN region, the value in the CMS + AFN 100 mg/kg group did not decline as markedly as that in the control group (Figure 4). Repeated-measures one-way ANOVA found an overall interaction: AVP (PVN) (F (3,12) = 8.27, *p* < 0.01), AVP (SON) (F (3,12) = 4.38, *p* < 0.05), and AVP (IHC) (F (3,12) = 8.84, *p* < 0.01).

#### 3.3.2. Expression of GR in the Hypothalamic PVN Region

**Figure 5:**
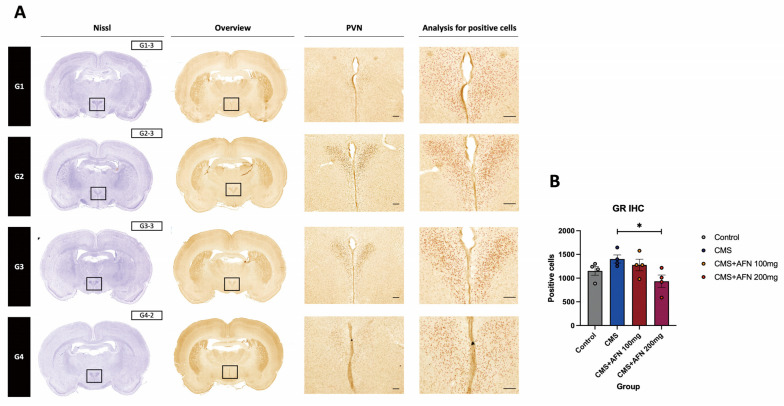
Quantification analysis of GR-positive cells in the hypothalamic PVN across different groups. GR-positive cells within the hypothalamic PVN region of each group were enumerated. These cells were visualized using IHC staining with GR antibody. (**A**) In the accompanying figure, GR-positive cells are identified as red dots. (**B**) The bar chart represents the counts of GR-positive cells in the PVN for each experimental group. Notably, the CMS + AFN 200 mg/kg group demonstrated a significant reduction in GR-positive cells compared to the CMS group. *, *p* < 0.05. The error bars represent mean ± SEM. Scale bar = 100 µm.

Maintaining a consistent negative feedback mechanism within the HPA is crucial for an appropriate stress response in animals. Upon detecting stress, the levels of AVP and CRF in the hypothalamus remained elevated. These hormones, in turn, stimulate the release of ACTH into the bloodstream, leading to increased secretion of glucocorticoids from the adrenal gland. Prolonged stress can result in excessive glucocorticoid levels in the bloodstream, potentially leading to resistance to glucocorticoids at the hypothalamic glucocorticoid receptor. As our results indicate, there was an increase in the number of cells expressing the GR in the CMS group. However, a significant decrease in GR expression was evident only in the CMS + AFN 200 mg/kg group. However, a significant decrease in GR expression was evident only in the CMS + AFN 200 mg/kg group. Contrarily, no significant increase in the number of GR-positive cells was observed between the control and CMS groups (*p* = 0.094). When interpreting these results via IHC, it is essential to consider them semi-quantitatively (Figure 5). Repeated-measures one-way ANOVA found an overall interaction: GR IHC (F (3,12) = 3.34, *p* = 0.05).

#### 3.3.3. c-fos Activition Patterns Post-AFN Administration in Stressed Animals

**Figure 6:**
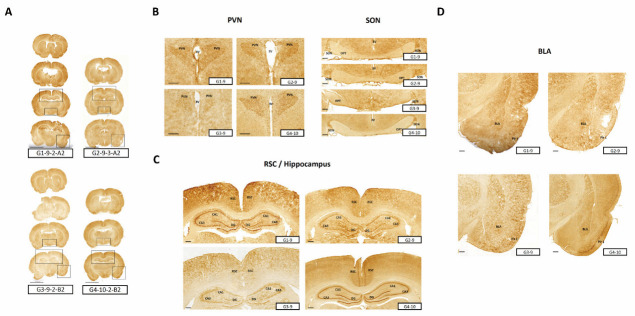
c-fos response in brain regions following AFN administration. Following a week of CMS exposure, animals were euthanized, and their brains were extracted 2 h after a single AFN application. Brain tissues from two mice per group were cryosectioned and subsequently subjected to immunohistochemistry (IHC) using a c-fos-specific antibody. Although c-fos responses across the groups were compared, observations from the G4 group were particularly noteworthy due to some tissue sectioning quality issues in other groups. Pronounced alterations in c-fos expression were detected in regions such as PVN, SON, BLA, piriform cortex 1 (Pir1), and retrosplenial cortex (RSC). For visualization, the scale bar in image (**A**) represents 5 mm, whereas those in images (**B**–**D**) correspond to 100 μm each.

Animals designated for c-fos response assessment were euthanized two hours following AFN administration after a week of stress exposure. Comprehensive coronal sections of the brain were obtained using a c-fos-specific antibody. Initially, the strategy was to dissect the cerebral hemispheres into coronal and sagittal sections; however, tissue conditions rendered sagittal sectioning unfeasible. Nevertheless, the brain regions activated by AFN were discerned primarily based on the tissues from the CMS + 200 mg/kg group, given their superior conditions for such an analysis. Notably, c-fos activation was predominantly observed in the PVN, SON, BLA, Piriform cortex 1 (Pir 1), and retrosplenial cortex (RSC) (Figure 6).

### 3.4. Quantitative Analysis of BDNF and CRF Expression in the Hypothalamus via Western Blot

**Figure 7:**
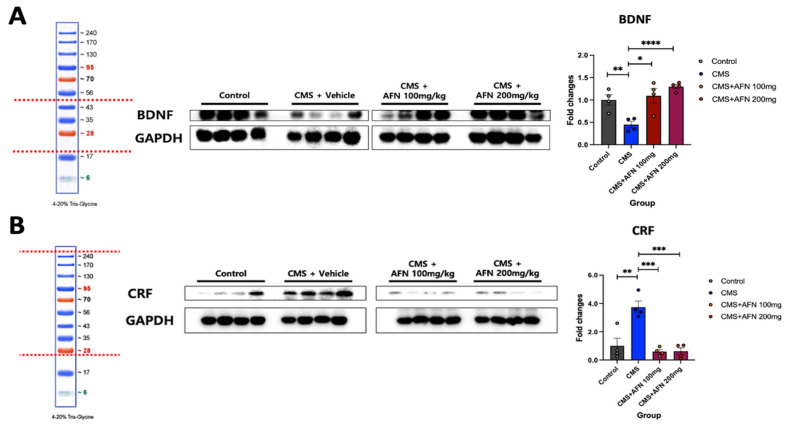
Comparison of expression levels of BDNF and CRF proteins in the hypothalamus of each group. (**A**) Although the CMS with vehicle group displayed significantly reduced BDNF levels, the AFN-treated groups maintained BDNF levels comparable to the control group, showing a notable difference from the CMS with the vehicle group. (**B**) The impact of repeated oral administration of AFN on CRF expression in the hypothalamus under CMS was assessed using Western blot. The CMS group, subjected to repeated CMS with only vehicle administration, exhibited a substantial increase in CRF expression. In contrast, the AFN-administered groups retained CRF levels akin to the control group. ****, *p* < 0.001; ***, *p* < 0.005; **, *p* < 0.01; *, *p* < 0.05 between the groups. The error bars represent mean ± SEM.

As demonstrated in this study, it is evident that neuroplasticity in the hypothalamus, notably reduced by CMS, exhibited a significant dose-dependent increase with AFN treatment. Notably, the variation in the observed values in the CMS + AFN 200 mg/kg group was markedly lower than that in the CMS + AFN 100 mg/kg group (Figure 7A). In the hypothalamic samples, the CMS group exhibited a pronounced elevation in CRF protein expression compared to the control group. However, both AFN-treated groups (CMS + AFN 100 mg/kg and CMS + AFN 200 mg/kg) showed reduced CRF protein levels compared to the control group. Nevertheless, the differences between the two AFN doses were not statistically significant (Figure 7B). Repeated-measures one-way ANOVA found an overall interaction: BDNF (F (3,12) = 11.17, *p* < 0.005), and CRF (F (3,12) = 16.86, *p* < 0.005).

### 3.5. Plasma Levels of ACTH, CORT, and 5-HT Measured by ELISA Assay

**Figure 8:**
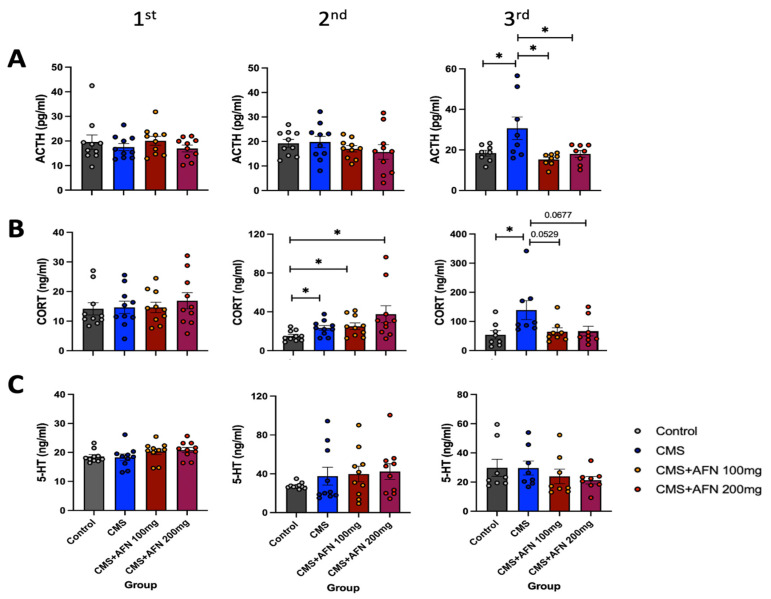
Measurement of ACTH, CORT, and 5-HT levels in peripheral blood from corresponding animal groups. (**A**) ACTH levels were measured at three different time points: before CMS initiation, one week post CMS, and after three weeks of AFN administration. A significant rise in ACTH was observed at the third collection, particularly in the group exposed solely to CMS with the vehicle for approximately four weeks. (**B**) Corticosterone (CORT) levels were evaluated until the second blood collection, before any AFN administration, and a marked elevation was observed compared to the control group (*, *p* < 0.05). Although repeated AFN dosing appeared to attenuate the stress response induced by the four-week CMS regimen, it did not reach statistical significance. Notwithstanding the lack of statistical significance, given the value was close to *p* < 0.05, it suggests that AFN administration might possess anti-stress properties. (**C**) The variation in measured serotonin values was initially minimal (1st 5-HT), but as the CMS regimen progressed, the spread of the measured values widened (2nd and 3rd 5-HT). This pattern persisted even in the AFN-administered groups, where no distinct trends in measured values emerged. *, *p* < 0.05 between the groups. The error bars represent mean ± SEM.

ACTH levels across all groups remained comparable until the first week after stress application. However, by the second week, a notable increase was observed in the CMS group. In contrast, the AFN group showed no significant increase. Notably, the variation in ACTH levels within the CMS group was substantial (Figure 8A). Repeated-measures one-way ANOVA found an overall interaction: ACTH 1st (F (3,36) = 0.60, *p* = 0.6212), ACTH 2nd (F (3,36) = 0.08, *p* = 0.500), ACTH 3rd (F (3,28) = 5.14, *p* < 0.01). Regarding CORT, a significant elevation was observed in the three groups subjected to CMS when compared with the control group at one week post stress. However, this increase exhibited a declining trend in the AFN-treated groups by the third week. Repeated-measures one-way ANOVA found an overall interaction: CORT 1st (F (3,36) = 0.31, *p* = 0.8205), CORT 2nd (F (3,36) = 3.53, *p* < 0.05), and CORT 3rd (F (3,28) = 3.55, *p* < 0.05). Statistical significance remained elusive despite the substantial dispersion of measured values across groups. However, it’s worth noting that the closeness of the *p*-values to 0.05 suggests the potential relevance of these findings (CMS + AFN 100 mg/kg; *p* = 0.0529 vs. CMS, and CMS + AFN 200 mg/kg; *p* = 0.0677 vs. CMS) (Figure 8B). The initial measurement of 5-HT exhibited minimal variance within or between the groups. In contrast, considerable disparities emerged in the values estimated during the second and third assessments. Consequently, statistical significance was not achieved due to the significant deviations in mean values between groups. Based on the outlined CMS schedule, a three-week AFN administration did not induce changes in 5-HT levels in the stress model (Figure 8C). Repeated-measures one-way ANOVA found an overall interaction: 5-HT 1st (F (3,36) = 1.65, *p* = 0.1955), 5-HT 2nd (F (3,36) = 0.76, *p* = 0.5255), and 5-HT 3rd (F (3,28) = 0.82, *p* = 0.4963).

## 4. Discussion

In contemporary society, concerns about stress, depression, anxiety disorders, and cognitive decline are escalating, affecting a growing number of individuals [33,34,35]. Our working group strives to find effective alternatives to address the stress and depression experienced by many individuals; the present study was designed as a continuation of our previous work [27]. In our comprehensive exploration of the therapeutic potential of saffron extract (AFN), we utilized a male Wistar rat model subjected to unpredictable chronic mild stress (CMS) to unravel its neuroprotective and anti-stress attributes. Our findings have woven a complex tapestry of insights, each contributing to our understanding of the intricate interplay between stress, neurochemical alterations, and the potential remedial effects of AFN. Numerous studies have underscored saffron’s potential as a therapeutic agent against stress and depression [15,23,26,36]. The increased risk of metabolic diseases stemming from psychological disorders has fueled a growing interest in AFN’s therapeutic potential [37,38]. The evaluation of the regulatory capacity of the HPA axis, in both humans and animals under specific conditions, has emerged as a crucial approach to assessing chronic stressors [3,29,30,31,39,40,41]. Despite a burgeoning body of research on AFN’s anti-stress properties, few studies have specifically investigated its role in normalizing the regulation of the HPA axis, a pivotal system for stress modulation in the body. Additionally, the interplay between enhanced brain plasticity, hormonal changes, and behavioral shifts following AFN administration, particularly in the context of accumulated stress, remains largely unexplored. 

In this context, our study sought to provide deeper insights into the specific stress-alleviating mechanisms of AFN. By identifying the brain regions directly and indirectly influenced by AFN, we aim to illuminate novel avenues for future research (Figure 6). An intriguing observation in our study was the phenomenon of reduced weight gain in the CMS-treated group. Although AFN treatment, especially at higher concentrations, partially mitigated this deceleration in weight gain, it was not accompanied by corresponding changes in food and water intake (Figure 1). The consistent food and water consumption across groups challenges the simplistic notion that reduced caloric intake is the primary driver of CMS-induced weight changes. Instead, this suggests the possibility of underlying metabolic or endocrinological adjustments mediated by stress, partially counteracted by AFN. This underscores AFN’s potential influence on metabolic pathways and energy homeostasis in response to stress. 

In the domain of the HPA axis, a central neuroendocrine system orchestrating our physiological stress response, CMS unequivocally disrupted this intricate system. The therapeutic potential of AFN was evident in our study, as it not only mitigated CMS- induced dysregulation but also exhibited a dose-dependent modulation of key markers. It reduced the expression levels of AVP, GR, and CRF in critical stress-processing brain regions. Glucocorticoid hormones, cortisol in humans and corticosterone (CORT) in rodents, produced by the HPA axis and working in tandem with neuropeptides and neurotransmitters, play a pivotal role in fostering resilience, enabling the organism to handle challenges and stress. Notably, CORT, the HPA axis’s final hormone, acts alongside an array of stress-responsive systems. These include the CRF/AVP and POMC pathways, along with various neurotransmitters, neuropeptides, and growth factors/cytokines. Our findings demonstrated that oral administration of AFN significantly reduced the expression of AVP (as shown in Figure 4) and CRF (as shown in Figure 7B) in the hypothalamus under CMS conditions. Based on these results, it is posited that the oral AFN administration led to a decrease in ACTH and CORT secretion (Figure 8A,B), which in turn contributed to a reduction in GR expression within the hypothalamic PVN. CORT functions by activating two receptors, GR and mineralocorticoid receptor (MR), which together act akin to an on/off switch, orchestrating daily rhythms, managing stress responses, and facilitating adaptation to changing conditions. Inducing overexpression of GR post weaning and during the animal’s lifespan did not lead to changes in affective responses or heightened emotional reactivity [42]. In contrast, MR overexpression, though less extensively researched, produced a unique phenotype characterized by reduced anxiety-like behaviors [43]. This supports the perspective that the balance between GR and MR is vital in regulating various cellular functions and behavioral outcomes [44]. However, unfortunately, due to the absence of data regarding the expression of MR in our study, we were unable to determine the relationship between CORT and MR expression. This restoration of HPA balance by AFN is not merely a theoretical observation. From a therapeutic perspective, it underscores AFN’s potential as a neurochemical modulator, which could be of paramount importance in stress-related disorders characterized by HPA axis dysregulation. 

Neuroplasticity, the brain’s capacity to adapt and remodel, has emerged as another focal point of interest. Our findings regarding BDNF, a cornerstone molecule in neuroplasticity, are compelling. The increase in BDNF levels, particularly under the influence of AFN, aligns with an emerging body of literature suggesting that enhanced neuroplasticity represents a robust counterstrategy against stress-induced neuronal dysfunction and atrophy. Given that reduced neuroplasticity is often implicated in various neuropsychiatric conditions, the effects of AFN cannot be underestimated. Our investigation into blood biochemistry served a dual purpose: to validate our findings and broaden our understanding of the systemic effects of AFN. Although the suppression of ACTH and CORT levels supported our neurochemical findings, the impact on 5-HT was more intricate. The absence of significant alterations in 5-HT levels despite AFN administration suggests a nuanced interaction between AFN and the serotonergic system, implying that the benefits of AFN may manifest through mechanisms beyond serotonergic modulation. 

The SPT results add another dimension. These findings reveal that while AFN partially ameliorated CMS-induced anhedonia, it did not fully restore it. This raises intriguing questions about the breadth and limitations of AFN’s therapeutic effects. Indeed, our previous results revealed an antidepressant effect in the SPT outcomes. However, these findings diverge from the intended design of the current study. Considering the high susceptibility of rodents to stress and its potential to precipitate a profound decrease in social behaviors [45], there is an undeniable risk that such variations could substantially affect the study’s conclusions. The design of this study incorporated an ancillary protocol for the collection of biological samples beyond the CMS model to procure biochemical markers from the subjects. This unavoidable additional process may have inadvertently subjected the animals to an allostatic overload by introducing additional stressors that exceed their capacity to maintain stress resilience or allostasis well. 

Lastly, the potential activation of specific brain regions by AFN, namely PVN, SON, BAL, RSC, and Pir1, complements our overarching narrative. Given their well-established roles in stress perception and processing, these regions suggest a broader and more comprehensive cerebral impact of AFN. The PVN, SON, and BAL regions, both in our research and in previous studies, are well documented as primary areas responsive to psychological stress [46,47]. The close association of the Pir1 region with the brain’s learning capabilities and mood-related disorders provides insights into potential brain functions activated by AFN [48,49]. The hippocampus and the RSC share a direct anatomical link, and both play pivotal roles in cognition and memory processes [50]. Previous studies have demonstrated that RSC appears to be more sensitive to signals predicting rewards or necessitating specific actions [50]. In contrast, the hippocampus exhibits distinct neuronal responses in different contexts [51], suggesting that it focuses on deciphering various scenarios, whereas the RSC is more responsive to cues with immediate behavioral significance.

In summary, as previously mentioned, it is evident that organisms not only strive for resilience in the face of stress from both internal and external stimuli but also initiate physiological alterations to acclimate to stress via CORT secretion. The chronic oral administration of AFN is anticipated to substantially enhance the animals’ capability to withstand and recuperate from CMS-induced stress, along with fostering physiological adaptability (allostasis). Furthermore, the recurrent administration of AFN may offer a promising alternative, particularly when stress becomes prolonged or when it surpasses the organism’s adaptive threshold (allostatic overload). Remarkably, it was confirmed that oral administration of AFN showed a dose-dependent stress-reducing effect in almost all measured data other than the results of SPT or 5-HT in countering CMS stress. AFN holds promise as a potent modulator of stress-induced neurochemical imbalances. Its effects on the HPA axis, neuroplasticity markers, and depression-like behaviors are scientifically intriguing and could potentially pave the way for novel therapeutic avenues. Nevertheless, the road ahead calls for more nuanced, detailed, and possibly multimodal investigations, particularly regarding safety profiles, interactions with the serotonergic system, and broader systemic effects.

## 5. Conclusions

In this study, we explored the potential therapeutic benefits of saffron extract (AFN) using Wistar rats exposed to unpredictable CMS. Notably, this extract demonstrated significant neuroprotective and remedial effects in the face of stress-induced disruptions. The administration of AFN, particularly at higher concentrations, effectively regulated the HPA axis and enhanced markers associated with neuroplasticity. Therefore, chronic oral AFN administration is posited to be a highly effective strategy for enhancing stress resilience and facilitating recovery from allostatic overload in CMS animal models, offering a potential therapeutic avenue for stress-related disorders. Nevertheless, its influence on 5-HT levels and sucrose preference seems to be limited, suggesting that its effects may be selective or dependent on specific conditions for their full realization. These findings shed light on the path for further in-depth investigations into AFN’s mechanisms of action and its potential as a therapeutic intervention for stress-related disorders.

## Figures and Tables

**Table 1:** Unpredictable chronic mild stress (CMS) regime.

	Weeks			
	1st Week (No-Drug)	2nd Week	3rd Week	4th Week
Monday	RFA ^1^ 2 h	RFA 2 h	RFA 2 h	RFA 2 h
Tuesday	EWB ^2^ 5 h	EWB 5 h	EWB 5 h	EWB 5 h
Wednesday	FD ^3^ 16 h	FD 16 h	FD 16 h	FD 16 h
Thursday	NB ^4^ 16 h	NB 16 h	NB 16 h	NB 16 h
Friday	CT ^5^ 2 h	CT 2 h	CT 2 h	CT 2 h
Saturday	WD ^6^ 16 h	WD 16 h	WD 16 h	WD 16 h
Sunday	CT ^7^ 2 h	CT 2 h	CT 2 h	CT 2 h

^1^ Restricted food access, ^2^ empty water bottle, ^3^ food deprivation, ^4^ no bedding, ^5^ cage tilting, ^6^ water deprivation, ^7^ cage tilting.

## Data Availability

The data presented in this study were limited to requests from the corresponding author.
